# Effect of the Duration of Deep Hypothermic Circulatory Arrest on the Neurodevelopmental Outcomes in Children Undergoing Cardiac Surgery

**DOI:** 10.3390/pediatric16030063

**Published:** 2024-08-31

**Authors:** Abdullah H. Ghunaim, Basma Aljabri, Ahmed Dohain, Ghassan S. Althinayyan, Abdulaziz I. Aleissa, Ahmad T. Alshebly, Rayan A. Alyafi, Tareg M. Alhablany, Ahmed M. Nashar, Osman O. Al-Radi

**Affiliations:** 1 Cardiac Surgery Division, Department of Surgery, King Abdulaziz University, Jeddah 21589, Saudi Arabia; ghunaim.ah@gmail.com (A.H.G.); oradi@kau.edu.sa (O.O.A.-R.); 2 Faculty of Medicine, King Abdulaziz University, Jeddah 21589, Saudi Arabia; galthinayyan@gmail.com (G.S.A.); abdulazizaleissa30@gmail.com (A.I.A.); dralshebly@gmail.com (A.T.A.); rayanyafi@gmail.com (R.A.A.); tareqalhablany@gmail.com (T.M.A.); ahmednashar96@gmail.com (A.M.N.); 3 Department of Pediatrics, King Abdulaziz University, Jeddah 21589, Saudi Arabia; 4 Pediatric Cardiology Division, Department of Pediatrics, Cairo University, Cairo 12613, Egypt; adohain@yahoo.com; 5 Pediatric Cardiology Division, Department of Pediatrics, King Abdulaziz University Hospital, Jeddah 21589, Saudi Arabia

**Keywords:** Bayley Scales, congenital heart diseases, deep hypothermic circulatory arrest, neurodevelopmental

## Abstract

**Background/Objectives:** Deep hypothermic circulatory arrest (DHCA) is safe, but subtle neurodevelopmental deficits may persist far beyond the perioperative period. We aimed to investigate the relationship between DHCA duration and neurodevelopmental outcomes in young children undergoing cardiac surgery with DHCA. **Methods:** Children aged < 42 months, including neonates who underwent cardiac surgery using DHCA without regional perfusion techniques, were included as the DHCA group. Children in the same age range who underwent cardiac surgery without DHCA were included as the control group. All enrolled patients underwent neurodevelopmental assessment using the Bayley Scales of Infant and Toddler Development (BSTID) by a trained pediatrician, and 17 DHCA patients and 6 control patients completed the BSTID assessment. **Results:** Both groups showed no significant preoperative, operative, or postoperative differences. Adjusted multivariable analysis revealed that prematurity and age at assessment were significant changing predictors of each of the BSTID components (*p* < 0.001), except for the gross motor component, where only age at assessment was a significant adjusting predictor. Longer DHCA was associated with lower fine and gross motor BSTID components; however, the association was not statistically significant (*p* = 0.06). **Conclusions:** Long-duration DHCA without regional perfusion techniques may be associated with less optimal neurodevelopmental outcomes.

## 1. Introduction

Neurodevelopmental delay is the most common complication in patients undergoing congenital cardiac surgery [1], accounting for approximately 25–50% of all disabilities of patients surviving surgery [2]. This consists in a delay in fine motor, gross motor, social, speech, and language skills, as well as detrimental effects on cognitive function [3]. The delay is due to complex preoperative, intraoperative, and postoperative factors [4].

Congenital heart disease (CHD) is the most common birth defect globally, accounting for approximately 6–11 cases in every 1000 births [4]. In Saudi Arabia, similar numbers were documented in a systematic review published in 2015 [5]. Advances in CHD management have improved vastly in the past few decades, leading to a decrease in the mortality rate of patients undergoing congenital cardiac surgery [3,4]. However, neurodevelopmental complications in survivors were documented, which can be linked to many factors that could affect the outcome of the surgery, including the age of patients at repair, length of intensive care unit (ICU) stay, and use of cardiopulmonary bypass (CPB) and circulatory arrest [1].

The support technique used is a critical intraoperative source of morbidity in patients with CHD who undergo surgery. Two standard techniques are deep hypothermic circulatory arrest (DHCA) and low-flow CPB (LFCPB). Both techniques have made many complex surgical repairs feasible and have led to a decrease in the mortality rate in patients with CHD [3,4]. However, the literature indicates that prolonged periods of DHCA and LFCPB are associated with a high risk of development of future neurological injury [6,7].

Neurodevelopmental delay affects the quality of life and imposes a burden on both the patients and their families [1]. Although the recent literature has established a significant relationship between congenital cardiac surgery and neurodevelopmental complications [1], no clear connection between the duration of DHCA and LFCPB and neurodevelopmental outcomes was found.

In this study, we aimed to investigate the relationship between DHCA duration and neurodevelopmental outcomes in young children undergoing cardiac surgery with DHCA in order to understand its parameters and thus provide patients with better neurodevelopmental outcomes.

## 2. Materials and Methods

### 2.1. Sample

After obtaining ethics approval from the Biomedical Ethics Unit at King Abdulaziz University, we retrospectively enrolled patients who underwent CHD surgery with DHCA (DHCA group) or without DHCA (control group) at a single center between January 2015 and December 2018.

**Figure 1:**
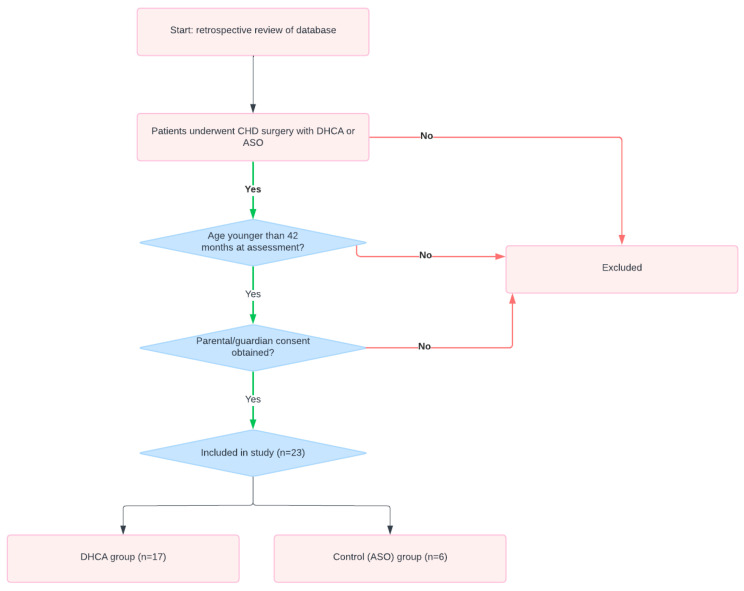
Flowchart of the recruitment process of the study showing patients who underwent CHD surgery with DHCA or ASO. CHD, congenital heart disease; DHCA, deep hypothermic circulatory arrest; ASO, arterial switch operation.

Using a retrospective review of a prospectively maintained database, we identified patients aged younger than 42 months who underwent congenital cardiac surgery and included those whose parents/guardians consented to take part in the study and to a visit for assessment of the child. We excluded those who were older than 42 months at the time of assessment and those whose parents/guardians did not provide consent. Twenty-three patients were included (DHCA group, *n* = 17; control group, *n* = 6) (Figure 1).

### 2.2. Data Collection

Data collected included demographic characteristics, height, and weight at surgery and at the time of the last follow-up, procedures performed, associated comorbidities, type of surgery performed, duration of ICU stay, duration of hospital stay, duration of CPB, duration of DHCA, early and late complications, and mortality.

### 2.3. Neurodevelopmental Assessment

After obtaining these data, we contacted the patients’ families and brought them to the neurodevelopmental clinic at our center. After receiving the guardians’ written consent, we performed an assessment using the Bayley Scales of Infant and Toddler (BSTID), Third Edition (Bayley-III), which is a well-known screening tool for measuring the risk of developing neurodevelopmental delay in infants and toddlers from age 1 month to 42 months and yields cognitive, communicative, and motor composite scores [8]. The assessment was performed by a developmental pediatric consultant who is well-experienced in Bayley-III. Personnel performing the test were blinded to group allocation.

### 2.4. Statistical Analyses

All statistical analyses were performed using Stata version 16.1 (Stata Corp, College Station, TX, USA). A two-tailed *p*-value < 0.05 was considered statistically significant. Categorical variables are presented as frequencies, and continuous variables are presented as a mean and standard deviation or median.

#### 2.4.1. Data Presentation and Group Comparison

We compared the preoperative, operative, and postoperative variables between patients in the DHCA (*n* = 17) and control (*n* = 6) groups. The Shapiro–Wilk test was performed to assess the distribution of the continuous variables. Normally distributed continuous variables were compared using Student’s *t*-test and are presented as a mean and standard deviation. Non-normally distributed data were compared using the Mann–Whitney U test and are presented as a median [25th–75th percentiles]. Binary and nominal variables were compared using the chi-square test or Fisher’s exact test if the expected frequency was less than 5 and are presented as frequencies and percentages.

#### 2.4.2. Linear Regression Analysis

Univariable linear regression analysis was performed to assess factors associated with cognition, receptive communication, expressive communication, fine motor skill, and gross motor skill scores. Variables with a *p*-value < 0.2 in the univariable model were included in a multivariable linear regression model. Backward elimination was performed in the multivariable linear regression model for variables with a *p*-value > 0.1; hence, the final model only included variables with a *p*-value ≤ 0.1. The Breusch–Pagan test was used to assess heteroskedasticity, and the distribution of the residual was evaluated to confirm a normal distribution. Multicollinearity was tested using the variance inflation factor. Spearman’s correlation was used to assess the correlation between scores and DHCA duration.

## 3. Results

### 3.1. Preoperative Data

We enrolled 35 patients, of whom 17 of 25 DHCA patients and 6 of 10 control patients completed the BSTID assessment. The patients who did not undergo the evaluation were well, asymptomatic, and their parents/guardian declined the evaluation. Age, sex, weight, genetic syndrome, prenatal diagnosis, mode of delivery, socioeconomic status, or parental education did not differ between the groups. None of the patients’ siblings had neurodevelopmental delay. No patient had a preoperative stroke or prenatal asphyxia. One patient in the DHCA group had undergone previous bilateral hernia repair (Table 1).

### 3.2. Operative and Postoperative Data

All patients in the control group had undergone an arterial switch operation. In the DHCA group, the surgeries performed were stage I Norwood–Sano (*n* = 4), aortic coarctation and ventricular septal defect repair (*n* = 10), interrupted aortic arch repair (*n* = 2), and arterial switch (*n* = 1). The CPB duration, excluding the DHCA duration, was longer in the control group than in the DHCA group (101 [74–123] vs. 61 [56–72] minutes, *p* = 0.002). If the DHCA time was added to the CPB time, the difference between the two groups was not significant. No other operative or postoperative differences were statistically significant. The postoperative outcomes did not differ significantly between groups. No in-hospital mortality was reported in either group (Table 2).

### 3.3. Neurodevelopmental Assessment

**Figure 2:**
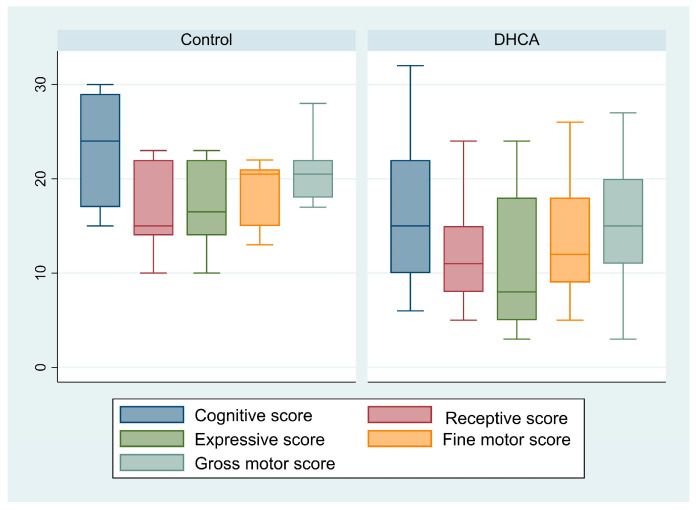
Box plots comparing the cognitive, receptive communication, expressive communication, fine motor, and gross motor scores between groups. DHCA, deep hypothermic circulatory arrest.

Neurodevelopmental assessment using BSTID was performed in a median time of 717 [318–1061] days after surgery, with no difference between groups (*p* = 0.43). There were no differences in age at the time of assessment or in the scales’ component between groups (Table 3, Figure 2).

### 3.4. Factors Affecting Neurodevelopment

DHCA was non-significantly associated with a lower cognitive score (*p* = 0.08), and the score was negatively correlated with the DHCA duration, but this did not reach statistical significance (*p* = 0.39). Similarly, DHCA was associated with a lower expressive communication score but did not reach statistical significance (*p* = 0.06) (Table 4).

The fine and gross motor scores tended to be more affected in the DHCA group, but without statistical significance (*p* = 0.08 and *p* = 0.06, respectively) (Table 4). Increased duration of DHCA was associated with lower fine (*p* = 0.26) and gross motor (*p* = 0.18) components of the BSTID; however, the association failed to reach the preset level of statistical significance (Figure 2).

In the adjusted multivariable assessment, prematurity and age at assessment were significant changing predictors of each of the components of BSTID (*p* < 0.001), except for the gross motor component, for which only age at assessment was a significant predictor.

## 4. Discussion

Despite widespread recognition of a higher occurrence of neurodevelopmental delay among children with CHD and the need for neuroprotective strategies, little evidence is available for the optimal intraoperative perfusion strategy [9,10]. We investigated the relationship between DHCA duration and neurodevelopmental outcomes in young children undergoing cardiac surgery with DHCA. In the adjusted multivariable analysis, prematurity and age at assessment were significant changing predictors of each of the BSTID components (*p* < 0.001), except for the gross motor component, in which only age at assessment was a significant predictor. Longer DHCA was associated with lower fine and gross motor BSTID components scores, but without statistical significance (*p* = 0.06).

Many pediatric cardiac surgeons have been using DHCA, owing to its obvious technical advantages and the ability to ameliorate the morbidity associated with CPB, particularly in the smallest patients and neonates [9]. However, previous studies on the impact of DHCA on neurodevelopmental outcome have yielded conflicting findings. A prospective randomized study from the University of Michigan compared the use of DHCA with the use of regional cerebral perfusion and found no statistically significant difference in developmental testing results at the age of 1 year. Consistent with previous reports, they found the psychomotor development index (PDI) to be significantly more affected than the mental development index (MDI) [11,12]. Kosiorek et al. found a significant worsening of neurological outcomes in patients undergoing CHD surgery without DHCA [13]. Moreover, a study that used electroencephalograms (EEGs) to monitor patients concluded that seizures were increased in this group, EEG seizures were more common than clinical seizures, and seizures were associated with increased neurodevelopmental delay and neurological problems [13].

For patients with hypoplastic left-heart syndrome and other single-ventricle lesions, a retrospective study by Visconti et al. demonstrated no benefit of regional low-flow cerebral perfusion relative to DHCA regarding the neurodevelopmental outcomes at the age of 1 year [14]. Fuller et al. showed that CPB and DHCA durations were not predictive of developmental outcomes at the age of 1 year after infant cardiac surgery [15]. In the present cohort, DHCA was associated with lower cognitive, expressive communication, fine motor, and gross motor scores, but the associations failed to reach the preset level of statistical significance (*p* = 0.08, *p* = 0.06, *p* = 0.08, and *p* = 0.06, respectively), which could be due to the small number of patients included in the control group.

In contrast, earlier studies demonstrated that children who underwent DHCA had lower PDI scores at the age of 1 year and had impairment in gross and fine motor skills as well as speech apraxia at the age of 4 years [2,6]. Extended periods of DHCA were found to be associated with neurological damage resulting in adverse neurodevelopmental outcomes [2,16]. Hickey observed a significant correlation of the duration of circulatory arrest with lowered PDI and higher neurological abnormalities at the age of 1 year [17]. However, a 4-year assessment demonstrated that patients who underwent LFCPB and circulatory arrest did not differ in neurological examination findings or IQ, but the duration of circulatory arrest affected several subscores of cognitive function [17].

In the Single Ventricle Reconstruction trial [18], neither DHCA use nor duration was an independent risk factor for neurodevelopment outcomes at the age of 14 months, whereas an earlier study showed that neither the use nor the duration of DHCA was correlated with a worse outcome for PDI or MDI at the age of 1 year after infant cardiac surgery [15].

Consistent with the current study, Wypij et al. found that the effect of duration of DHCA on neurodevelopmental outcomes was nonlinear. Moreover, they showed that important late neurodevelopmental outcomes, such as fine motor function, academic achievement, and speech, were generally unaffected when a shorter duration of DHCA was used [16].

This discrepancy in the results is likely multifactorial and may be due to differences in cardiac diagnoses, perioperative and intraoperative support strategies, and patient-related risk factors, such as gestational age, sex, and ethnicity. Importantly, more recent studies included considerably different intraoperative support techniques and postoperative management facilities [15]. In the present cohort, despite an exhaustive list of perioperative and patient-related risk factors, adjusted multivariable analyses identified only prematurity as a risk factor for worsened scores on components of the BSTID (*p* < 0.001), except for the gross motor function.

Beca et al. reported that the severity of brain immaturity at birth is a predictor of the severity of neurodevelopmental impairment at the age of 2 years after infant cardiac surgery [19]. Additionally, CHD may alter brain development and increase vulnerability to hemodynamic instability perioperatively and to brain injury due to hypoxia and ischemia intraoperatively [1]. A previous report showed that preoperative risk factors of low gestational age and elevated preoperative lactate could classify 84% of mentally and/or motor-delayed children after cardiac surgery [15]. A recent study identified sex, race, birth weight, CHD type, genetic anomalies, and maternal education as important independent predictors of neurodevelopmental outcomes [1].

Our study had some limitations, including a small sample size, inherent limitations and the risk of bias of a retrospective study design (which limited genetic assessment in our patients), the potential for selection bias, a lack of generalizability to different populations, and the inclusion of preterm infants.

## 5. Conclusions

The use of DHCA without regional perfusion techniques may be associated with less optimal neurodevelopmental outcomes if the duration of DHCA is long. Future studies with larger sample sizes are needed to delineate the level of significance and length of the period of safe DHCA. Additionally, further studies should investigate safer alternatives to DHCA.

## Figures and Tables

**Table 1:** Preoperative patients’ characteristics.

	**Total Patients (*n* = 23)**	**Control (*n* = 6)**	**DHCA (*n* = 17)**	***p*-Value**
Sex (male)	15 (65.22)	5 (83.33)	10 (58.82)	0.37
Age at intervention (days)	21 [9–82]	6 [3–35]	31 [14–103]	0.05
Weight at intervention (kg)	3.2 [2.5–3.6]	3.5 [2.9–3.7]	2.8 [2.5–3.6]	0.40
Down syndrome	1 (4.35)	0	1 (5.88)	>0.99
Prenatal diagnosis	5 (21.74)	3 (50)	2 (12.5)	0.10
Preterm labor	2 (8.7)	0	2 (11.76)	>0.99
Socio-economic status				0.50
Low	12 (57.14)	4 (66.67)	8 (53.33)	
Middle	7 (33.33)	1 (16.67)	6 (40)	
Upper middle	2 (9.52)	1 (16.67)	1 (6.67)	
Father’s education				0.13
Primary school	1 (4.76)	0	1 (6.67)	
Secondary school	10 (47.62)	1 (16.67)	9 (60)	
Bachelor’s degree	9 (42.86)	4 (66.67)	5 (33.33)	
Master’s degree	1 (4.76)	1 (16.67)	0	
Mother’s education				0.32
Primary school	1 (4.76)	0	1 (6.667)	
Middle school	5 (23.81)	2 (33.33)	3 (20)	
Secondary school	6 (28.57)	0	6 (40)	
Bachelor’s degree	7 (33.33)	3 (50)	4 (26.67)	
Master’s degree	2 (9.52)	1 (16.67)	1 (6.67)	
Delivery mode				0.32
Vaginal	16 (69.57)	3 (50)	13 (76.47)	
Cesarean	7 (30.43)	3 (50)	4 (23.53)	
Previous non-cardiac surgery	1 (4.35)	0	1 (5.88)	>0.99
Preoperative ventilation (days)	0 [0–3]	0 [0–3]	2 [0–3]	0.56

Continuous data are presented as median [25th–75th percentiles], and categorical data are presented as numbers and percentages. DHCA, deep hypothermic circulatory arrest.

**Table 2:** Operative and postoperative data.

	Total Patients (*n* = 23)	Control (*n* = 6)	DHCA (*n* = 17)	*p*-Value
Duration of CPB (minutes)	68 [56–78]	101.5 [74–123]	61 [56–72]	0.002
Cross-clamp time (minutes)	45.57 ± 16.52	61.67 ± 18.59	39.88 ± 11.65	0.003
Highest creatinine (μmol/L)	55 [46–87.8]	54.5 [49–70]	68 [42–91]	0.93
Highest lactate (mmol/L)	4.67 ± 0.48	4.87 ± 1.14	4.6 ± 0.53	0.81
Postoperative acidosis	17 (73.91)	3 (50)	14 (87.5)	0.10
Postoperative hypoxia	11 (47.83)	4 (66.67)	7 (41.18)	0.37
Mechanical ventilation (days)	6 [4–8]	5 [2–7]	7 [4–12]	0.29
ECMO	1 (4.35)	0	1 (5.88)	>0.99
Open sternum	18 (78.36)	4 (66.67)	14 (82.35)	0.58
Open-chest duration (days)	2 [1.5–3]	3 [1.5–4]	2 [1.5–3]	0.53
Postoperative seizures	1 (4.35)	1 (16.67)	0	0.26
Pulmonary hemorrhage	4 (17.39)	1 (16.67)	3 (17.65)	>0.99
Lowest Ca level (mg/dL)	2.04 [1.88–2.14]	1.98 [1.55–2.12]	2.05 [1.93–2.14]	0.34
ICU stay (days)	12 [7–18]	9 [5–12]	15 [7–23]	0.13
Hospital stay (days)	16 [12–26]	11.5 [10–14]	19 [15–46]	>0.99
Surgical re-exploration	2 (8.7)	0	2 (11.76)	>0.99
Blood stream infection	6 (26.09)	0	6 (35.29)	0.14
Surgical site infection	2 (8.7)	2 (33.33)	0	0.06
Heart block	5 (21.74)	1 (16.67)	4 (23.53)	>0.99
Chest drain > 5 days	2 (8.7)	0	2 (11.76)	>0.99

Continuous data are presented as mean ± standard deviation or median [25th–75th percentiles], and categorical data are presented as numbers and percentages. Ca, calcium; CPB, cardiopulmonary bypass; DHCA, deep hypothermic circulatory arrest; ECMO, extracorporeal membrane oxygenation; ICU, intensive care unit.

**Table 3:** Neurodevelopmental assessment.

	Total Patients (*n* = 23)	Control (*n* = 6)	DHCA (*n* = 17)	*p*-Value
Age at assessment (days)	774.78 ± 343.56	865 ± 184.36	742.94 ± 284.12	0.47
Cognitive score	18.26 ± 7.99	23.17 ± 6.62	16.53 ± 7.87	0.08
Cognitive				0.49
At-risk	9 (39.13)	1 (16.67)	8 (47.06)	
Emerging	8 (34.78)	3 (50)	5 (29.41)	
Competent	6 (26.09)	2 (33.33)	4 (23.53)	
Receptive communication score	14 [8–15]	15 [14–22]	11 [8–15]	0.17
Receptive communication				>0.99
At-risk	6 (26.09)	1 (16.67)	5 (29.41)	
Emerging	10 (43.48)	3 (50)	7 (41.18)	
Competent	7 (30.43)	2 (33.33)	5 (29.41)	
Expressive communication score	12.43 ± 6.91	17 ± 4.90	10.82 ± 6.89	0.06
Expressive communication				0.49
At-risk	9 (39.13)	1 (16.67)	8 (47.06)	
Emerging	8 (34.78)	3 (50)	5 (29.41)	
Competent	6 (26.09)	2 (33.33)	4 (23.53)	
Fine motor score	14.91 ± 6.03	18.67 ± 3.72	13.59 ± 6.21	0.08
Fine motor				0.46
At-risk	5 (29.41)	0	5 (29.41)	
Emerging	12 (52.17)	4 (66.67)	8 (47.06)	
Competent	6 (26.09)	2 (33.33)	4 (23.53)	
Gross motor score	16.26 ± 7.21	21 ± 3.90	14.59 ± 7.43	0.06
Gross motor				0.05
At-risk	9 (39.13)	0	9 (52.94)	
Emerging	7 (30.43)	3 (50)	4 (23.53)	
Competent	7 (30.43)	3 (50)	4 (23.53)	

Continuous data are presented as mean ± standard deviation or median [25th–75th percentiles], and categorical data are presented as numbers and percentages. DHCA, deep hypothermic circulatory arrest.

**Table 4:** Factors affecting the cognitive, receptive communications, expressive communications, fine motor, and gross motor scores.

	Univariable		Multivariable	
	Coefficient (95% CI)	*p*-Value	Coefficient (95% CI)	*p*-Value
Cognitive score *				
Age at assessment (months)	0.53 (0.32 to 0.74)	<0.001	0.59 (0.45 to 0.74)	<0.001
Preterm	−9.05 (−20.94 to 2.85)	0.13	−13.38 (−19.22 to −7.53)	<0.001
CPB duration	0.11 (−0.02 to 0.24)	0.09	−	
DHCA	−6.64 (−14.13 to 0.86)	0.08	−	
Receptive communication score **				
Age at assessment (months)	0.33 (0.16 to 0.50)	0.001	0.38 (0.25 to 0.50)	<0.001
Preterm	−8.24 (−16.45 to 0.03)	0.049	−11 (−15.95 to −6.06)	<0.001
CPB	0.07 (−0.02 to 0.17)	0.12	−	
DHCA	−4.03 (−9.52 to 1.46)	0.14	−	
Expressive communication score ***				
Age at assessment (months)	0.39 (0.17 to 0.6)	0.001	0.44 (0.26 to 0.61)	<0.001
Preterm	−8.14 (−18.38 to 2.09)	0.11	−11.35 (−18.3 to −4.4)	<0.001
Down syndrome	−9.84 (−24.21 to 4.49)	0.17	−	
CPB time	0.10 (−0.01 to 0.21)	0.09	−	
DHCA	−6.18 (−12.57 to 0.22)	0.06	−	
Fine motor score ****				
Age at assessment (months)	0.39 (0.22 to 0.55)	<0.001	0.42 (0.32 to 0.53)	<0.001
Male	−3.78 (−9.13 to 1.58)	0.16	−	
Preterm	−8.12 (−16.87 to 0.63)	0.07	−10.62 (−14.68 to −6.55)	<0.001
Down syndrome	−8.27 (−20.85 to 4.30)	0.19	−	
CPB	0.07 (−0.28 to 0.17)	0.15	−	
DHCA	−5.08 (−10.72 to 0.56)	0.08	−2.1 (−4.70 to −0.49)	0.10
Gross motor score *****				
Age at assessment (months)	0.48 (0.30 to 0.67)	<0.001	0.51 (0.39 to 0.64)	<0.001
Male	−4.78 (−11.13 to 1.58)	0.13	−10.85 (−15.82 to −5.87)	<0.001
Preterm	−7.95 (−18.71 to 2.81)	0.14	−	
Down syndrome	−10.73 (−25.63 to 4.18)	0.15	−	
CPB	0.10 (−0.01 to 0.22)	0.08	−	
DHCA	−6.41 (−13.09 to 0.27)	0.06	−3.04 (−6.22 to −0.14)	0.06

* R2 (cognitive score) = 0.80. ** R2 (receptive communications score) = 72. *** R3 (expressive communications score) = 0.63. **** R2 (fine motor score) = 0.84. ***** R2 (gross motor score) = 0.84. CPB, cardiopulmonary bypass; DHCA, deep hypothermic circulatory arrest.

## Data Availability

Available upon request.
